# Anti-Apoptotic and Anti-Inflammatory Role of Trans ε-Viniferin in a Neuron–Glia Co-Culture Cellular Model of Parkinson’s Disease

**DOI:** 10.3390/foods10030586

**Published:** 2021-03-11

**Authors:** Domenico Sergi, Alex Gélinas, Jimmy Beaulieu, Justine Renaud, Emilie Tardif-Pellerin, Jérôme Guillard, Maria-Grazia Martinoli

**Affiliations:** 1 Cellular Neurobiology, Department of Medical Biology, Université du Québec, Trois-Rivières, QC G9A 5H7, Canada; Domenico.sergi2@uqtr.ca (D.S.); alex.gelinas2@uqtr.ca (A.G.); jimmyb.92@hotmail.com (J.B.); Justine.renaud@uqtr.ca (J.R.); Emilie.tardif-pellerin@uqtr.ca (E.T.-P.); 2 Adelaide Medical School, The University of Adelaide, Adelaide, SA 5000, Australia; 3 UMR CNRS 7285 IC2MP, Equipe 5 Synthese Organique, UFR Médecine et Pharmacie, Université de Poitiers, 86073 Poitiers CEDEX 9, France; jerome.guillard@univ-poitiers.fr; 4 Department of Psychiatry and Neuroscience, U. Laval and CHU Research Center, Québec, QC G1V 4G2, Canada

**Keywords:** trans-ε-viniferin, resveratrol, neuroprotection, oxidative stress, dopamine, apotosis, neuroinflammation, Parkinson’s disease

## Abstract

The polyphenol trans-ε-viniferin (viniferin) is a dimer of resveratrol, reported to hold antioxidant and anti-inflammatory properties. The aims of our study were to evaluate the neuroprotective potential of viniferin in the nerve growth factor (NGF)-differentiated PC12 cells, a dopaminergic cellular model of Parkinson’s disease (PD) and assess its anti-inflammatory properties in a N9 microglia–neuronal PC12 cell co-culture system. The neuronal cells were pre-treated with viniferin, resveratrol or their mixture before the administration of 6-hydroxydopamine (6-OHDA), recognized to induce parkinsonism in rats. Furthermore, N9 microglia cells, in a co-culture system with neuronal PC12, were pre-treated with viniferin, resveratrol or their mixture to investigate whether these polyphenols could reduce lipopolysaccharide (LPS)-induced inflammation. Our results show that viniferin as well as a mixture of viniferin and resveratrol protects neuronal dopaminergic cells from 6-OHDA-induced cytotoxicity and apoptosis. Furthermore, when viniferin, resveratrol or their mixture was used to pre-treat microglia cells in our co-culture system, they reduced neuronal cytotoxicity induced by glial activation. Altogether, our data highlight a novel role for viniferin as a neuroprotective and anti-inflammatory molecule in a dopaminergic cellular model, paving the way for nutraceutical therapeutic avenues in the complementary treatments of PD.

## 1. Introduction

The incidence of neurodegenerative diseases is increasing worldwide, with Alzheimer’s disease (AD) and Parkinson’s disease (PD) being the most prevalent. Neurodegenerative diseases are characterized by neuronal loss, which in PD affects the dopaminergic neurons of the substantia nigra pars compacta (SNpc) [1]. Currently, apoptosis triggered by oxidative stress and neuroinflammation appears to be the main driver of dopaminergic neuron loss in PD [2].

Mitochondria are the principal source of reactive oxygen species (ROS), the mandatory bioproducts of oxidative metabolism. Mitochondrial dysfunction, a pivotal pathogenetic feature of PD, exacerbates oxidative stress by fostering ROS production which, in turn, via a feed-forward mechanism worsens mitochondrial dysfunction resulting in the activation of signaling cascades leading to apoptosis [3,4].

Due to their intrinsic low antioxidant activity and to the characteristic dopamine metabolism that generates pro-oxidant byproducts [5], dopaminergic neurons are particularly vulnerable to oxidative stress, which may explain their susceptibility to degeneration typically found in PD.

Besides oxidative stress, neuroinflammation represents another key mechanism responsible for neurodegeneration in PD [6]. In support of this, post mortem investigation in the brain of parkinsonian patients demonstrated elevated levels of activated microglia, and pro-inflammatory cytokines in the substantia nigra and in the striatum [7,8]. However, whether neuroinflammation is a cause or a consequence of neurodegeneration it remains a matter of debate. Nonetheless, in vitro cell co-culture models [9,10], have provided evidence that eliciting a pro-inflammatory phenotype in microglial cells using lipopolysaccharide (LPS), a well know pro-inflammatory molecule, promoted microglia-induced neuronal cell damage [9,11]. To the same extent, stereotaxic LPS injection into the brain of rodents can recapitulate neuroinflammation and promote dopaminergic neuron degeneration [12]. Thus, independently of whether neuroinflammation is involved in the etiology of PD or is the result of nigrostriatal pathway injury, sustained microglia pro-inflammatory activation can contribute to dopaminergic neuron loss.

Nowadays, strategies aimed at tackling oxidative stress and neuroinflammation may represent positive avenues to prevent neurodegeneration. In this regard, bioactive molecules derived from plants have emerged for their neuroprotective potential by countering oxidative stress, inflammation or both. Of these, resveratrol in light of its antioxidant [13] anti-inflammatory [11], and synergistic effects with other polyphenols such as quercetin [11,14,15], is considered one of the most promising. Nonetheless, resveratrol containing foods also present oligomeric forms of stilbenes whose biological effects remain to be fully elucidated, especially with regard to the central nervous system. Of these, ε-viniferin (viniferin), a naturally occurring resveratrol dimer with higher activity and stability, may contribute to the total amount of stilbenes consumed with foods [16]. This molecule shares several health promoting effects with resveratrol, including neuroprotective properties, as demonstrated in animal models of Huntington’s disease [17] and AD [18,19]. However, the effect of viniferin on oxidative stress and inflammation-induced injury in dopaminergic neurons remains to be elucidated.

Thus, the aim of this study was to determine whether viniferin was able to prevent 6-hydroxydopamine (6-OHDA)-induced cytotoxicity and apoptosis in a cellular model of PD, PC12 dopaminergic neurons. Furthermore, we aimed at elucidating whether this resveratrol dimer could prevent LPS-activated N9 microglial cells from inducing PC12 cell death in an N9 microglia PC12 cell co-culture system. The effect of viniferin on the aforementioned outcomes was also investigated in the presence of resveratrol, and resveratrol itself was used as reference molecule to benchmark the effect of viniferin. Our results show that viniferin can successfully protect PC12 dopaminergic neurons from oxidative stress-induced apoptosis, and is also able to prevent microglia from promoting neuronal death, with a synergetic effect being observed when resveratrol is used in a mixture together with viniferin.

## 2. Materials and Methods

### 2.1. Drugs and Chemicals

All reagents were purchased from Sigma-Aldrich (St. Louis, MO, USA) unless stated otherwise.

### 2.2. Extractions and Purifications of Polyphenols

Grape canes variety “Ugni” (Vitaceae) were collected from Cognac’s vineyards in France. Grape canes were extracted in an extruder (BC21 clextral) apparatus with a mixture of ethanol/water (8/2) as a solvent. The solution was concentrated after filtration, at 40 °C under a vacuum and lyophilized. Dried extract (4 g) was dissolved in 20 mL of a mixture called Biosolvants 1 with the following composition Makigreen D10–EtOAc–EtOH–H_2_O, (4:2:3:2) and injected into a centrifugal partition chromatography (CPC) apparatus. The CPC method used to obtain viniferin is the one presented previously [20], but using green solvents as Biosolvent 1 and Biosolvent 2 (Cyclopentane–EtOAc–EtOH–H_2_O, 1:2:1:2). Thus, fractions were collected and combined on the basis of HPLC analysis, providing a total of three fractions of interest. Fraction 1 (101 mg) corresponded to 95% pure resveratrol as white solid. Fraction 2 (175 mg) contained the dimer of interest, as with viniferin along with other compounds. Afterwards, fraction 2 was re-purified by semi-preparative HPLC and a yellow solid isolated was identified by 1H and 13C NMR spectroscopy analysis in acetone-d6 and mass spectrometry as with viniferin to 98% purity. The concentration of viniferin after extrusion–extraction is very dependent on the grape variety used but with Ugni’s grapecane the total polyphenol yield is 0.3% by weight with a concentration of viniferin in the extract grapine-shoot of between 15 and 20%.

### 2.3. Cell Culture and Treatments

**Figure 1:**
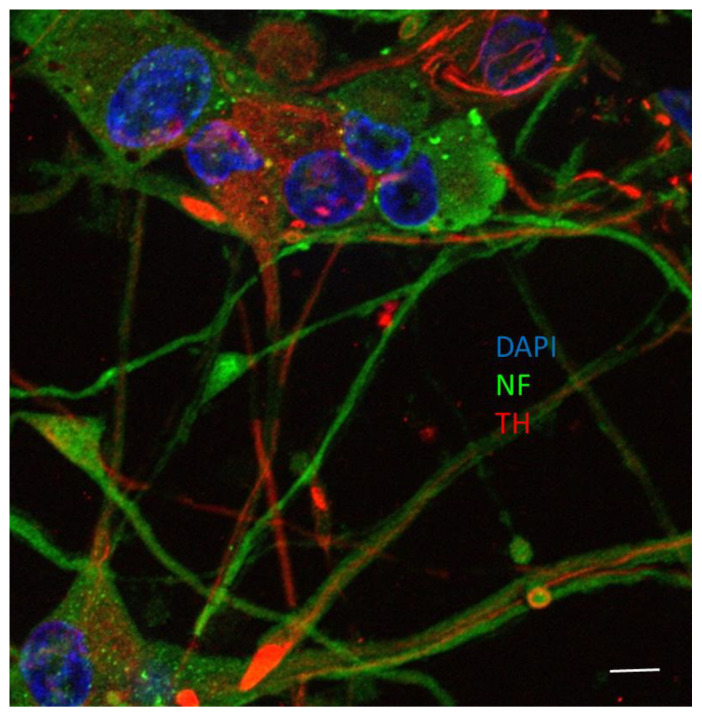
Representative microphotograph of nerve growth factor (NGF)-differentiated PC12 for 9 days by immunofluorescence. Nuclei are counterstained in blue with Dapi. NF: neurofilaments revealed with an anti-neurofilaments antibody (green). TH: tyrosine hydroxylase revealed with an anti-tyrosine antibody as a marker of dopamine (red). Scale bar = 10 µm.

A rat pheochromocytoma cell line (PC12 cells) was obtained from ATCC (Rockville, MD, USA). Cells were maintained in a humidified environment at 37 °C and 5% CO_2_ atmosphere and routinely grown in RPMI 1640 medium supplemented with 10% heat-inactivated horse serum (HS), 5% heat inactivated fetal bovine serum (FBS; Corning Cellgro, Manassas, VA, USA) and gentamicin (50 µg/mL). Neuronal differentiation was evoked by nerve growth factor-7S (NGF, 50 ng/mL) in RPMI 1640 supplemented with 1% FBS for 9–10 days, as already described [10,21]. NGF-differentiated PC12 cells (neuronal PC12) displayed a dopaminergic phenotype (Figure 1) as already reported [21,22]. They were pretreated with resveratrol or viniferin (generously provided by Dr. Jérôme Guillard, University of Poitiers, Poitiers, France) at 10–9 M or a mixture of the two molecules at 10–9 M for 3 h. Then 6-OHDA (50 µM) or LPS (2 μg/mL) [9] was added to the medium for 24 h. All experiments were performed in RPMI medium without phenol red, supplemented with 1% charcoal-stripped serum to remove steroids from the medium. Microglial cell line N9 was grown in Dulbecco’s modified Eagle’s medium nutrient mixture F12-Ham’s (DMEM-F12) supplemented with 10% HS.

### 2.4. Neuronal–Glial Co-Culture

Neuronal PC12 cells and N9 microglia were co-cultured without cellular contact to study the impact of LPS-activated microglia on the survival of neuronal cells, as we have already described [9]. In this co-culture system, microglial cells communicate with PC12 neuronal cells through a semipermeable membrane, in the absence of a direct contact between the two cellular systems [23]. Briefly, N9 microglial cells were grown onto culture inserts (pore size 0.4 μm; BD Falcon, Oakville, ON, Canada), LPS (2 μg/mL) was added. After 24 h, inserts containing N9 cells were washed with PBS and then transferred on neuronal PC12, for another 24 h. The PC12 supernatant was collected for cell death measurements with lactate dehydrogenase (LDH) cytotoxicity tests as described below. For immunofluorescent experiment, N9 microglial cells were grown in culture inserts and treated as described then transferred on neuronal PC12 cells grown previously on coverslips [9,11].

### 2.5. Cytotoxicity Measurements

Cytotoxicity was evaluated using a colorimetric assay based on the quantification of LDH activity released from damaged cells into the cell culture medium, as already described [20]. LDH is a stable cytoplasmic enzyme present in all cells, which is rapidly released into the cell culture supernatant upon plasma membrane damage. Enzyme activity in the cell culture medium is a direct function of lysed cells [24]. Briefly, 100 μL of cell-free culture media served to quantify LDH activity by measuring absorbance at 490 nm using a Thermo Lab Systems (Franklin, MA, USA). Total cellular LDH was determined cells lysed using 1% Triton X-100 (high control); the assay medium was used as a low control and was subtracted from all absorbance measurements:

Cytotoxicity (%) = (Experimental value-Low control)/(High control-Low control) × 100

### 2.6. MTT Assay

The cell metabolic activity was measured using 3-(4,5-dimethyltrazol-2-yl)-2,5-diphenyltetrazolium bromide (MTT) assay [21]. Neuronal PC12 cells, plated in 96-wells plates, were treated as already described and then incubated for 4 h at 37 °C with MTT dye (5 mg/mL), followed by solubilization in SDS 10% and the absorbance was measured at the 595 nm with a microplate reader (Thermo Lab Systems, Franklin, MA, USA).

### 2.7. Apoptosis- Specific DNA Denaturation Detection

Apoptosis-specific detection of DNA denaturation by formamide was assessed with the single-stranded DNA (ssDNA) apoptosis ELISA kit (Chemicon International, Telok Panglima Garang, Malaysia) as already described [15,22]. This procedure relies the on selective DNA denaturation by formamide in apoptotic cells, which does not occur in necrotic cells nor in cells with DNA damage in the absence of apoptosis [25]. The detection of denatured DNA was performed with a monoclonal antibody highly specific to ssDNA and a peroxidase-labelled secondary antibody on fixed neuronal PC12 cells. Following, ssDNA immune detection, the reaction was then terminated with a hydrochloric acid solution and ssDNA was quantified by measuring absorbance at 405 nm in a microplate reader (Thermolab Systems, Vasai-Virar, India). ssDNA was quantified relative to control conditions. Absorbance of positive (wells coated with provided ssDNA) and negative controls (wells coated with S1 nuclease that digest ssDNA) served as quality controls for the ELISA assay, as previously described [26,27].

### 2.8. Immunofluorescent Microscopy

Apoptotic P12 neuronal cells were detected by both terminal deoxynucleotidyl transferase dUTP nick end labeling (TUNEL, Roche Diagnostics, Basel, Switzerland) and cleaved caspase-3 immunofluorescence. Neuronal PC12 cells were seeded at 25,000 cells/cm^2^, differentiated and treated on collagen-coated coverslips in 24-well plates for immunofluorescent microscopy. Briefly, after viniferin and/or resveratrol and/or 6-OHDA treatments, cells were fixed in 4% paraformaldehyde for 15 min at 37 °C, then washed and incubated for 1 h at room temperature in a blocking and permeabilizing solution containing 1% BSA, 0.18% fish skin gelatin, 0.1% Triton X-100 and 0.02% sodium azide [22,26]. Cells were then incubated with anti-cleaved caspase-3 antibody (New England Biolabs, Pickering, ON, Canada) diluted 1:500, for 2 h at room temperature, followed by 90-min incubation with a Cy3-conjugated secondary antibody (Medicorp, Montreal, QC, Canada) diluted 1:500 for 1 h at 4 °C. The coverslips were then transferred to the TUNEL reaction mixture in a humidified atmosphere at 37 °C. The cells were rinsed with PBS, nuclei were counterstained in blue with DAPI for 10 min at 37 °C and mounted with ProLong Antifade kits (Invitrogen). PC12 neuronal cells were considered to be apoptotic when they were positive for cleaved caspase-3 and their nuclei were stained by TUNEL. The number of apoptotic neuronal cells among 300 randomly chosen neuronal cells was counted on 10 different optical fields from 3 slides per group [15], with Pro Express 6.3 software (Media Cybernetics, Rockville, MD, USA).

### 2.9. Electrophoresis and Immunoblot Analysis

Neuronal cells were grown and treated in 6-well plates. Total proteins were extracted with a Nuclear Extraction Kit (Active Motif, Carlsbad, CA, USA) and their concentration determined by using the BCA protein assay kit (Pierce Biotechnology Inc., Rockford, AZ, USA). Fifteen µg of protein were loaded onto a 12% SDS-polyacrylamide gel. After electrophoretic separation (125 V, for 1 h 30), proteins were transferred onto PVDF membranes (0.22 μm pore size, BioRad) at 25 V overnight. The membranes were blocked in TBST (TBS + Tween 0.05%) with 5% non-fat dry milk for 1 h at room temperature and incubated overnight at 4 °C with primary antibodies: anti-cleaved caspase-3 antibody (1:500) anti-cleaved PARP-1 antibody (1:500) or an anti-β-actin antibody (1:500) (Cell Signaling, Danvers, MA, USA). The blots were then rinsed three times with TBS 0.1% Tween 20 and incubated with peroxidase-conjugated (POD) secondary antibody (1:10,000) for 2 h at room temperature. Blots were washed with TBS 0.1% Tween 20 three times and the signal finally revealed by enhanced chemiluminescence with the AlphaEase FC imaging system (Alpha Innotech, San Leandro, CA, USA) and analyzed with AlphaEase FC software (Alpha Innotech) and ImageJ (imagej.nih.gov).

### 2.10. ELISA

Pro-inflammatory cytokines IL-1α and TNF-α were measured by specific ELISA kits (BioLegend, San Diego, CA, USA). Following pre-incubation with resveratrol, viniferin, or a mixture of the two molecules for 3 h, N9 microglial cells were administered with LPS 4 µg/mL for 24 h. Supernatants were collected after 27 h and processed for the presence of selected cytokines by ELISA, according to the protocols supplied by the manufacturer. Briefly, the supernatants were collected following the polyphenols and LPS challenges. 96-well plates were coated with mouse-specific monoclonal antibody (IL-1α and TNF-α) and after an overnight incubation, standards and samples were added to the wells for 2 h. The plates were then incubated in the presence of a biotinylated anti-mouse detection antibody for 1 h, followed by a 30 min incubation with an avidin horseradish peroxidase solution. Finally, a tetramethylbenzidine solution was added to the wells for 15 min in the dark to reveal the presence of the cytokines. The reaction was terminated by the addition of 2N sulfuric acid and resulting absorbance recorded at 450 nm using a microplate reader (Synergy H1, BioTek, Winnosky, VT, USA).

### 2.11. Statistical Analysis

Significant differences between groups were ascertained by One-way analysis of variance (ANOVA), followed by Tukey’s post-hoc analysis, performed using the GraphPad Prism8 for Windows (http://www.graphpad.com/). All data were expressed as means ± SEM from at least 3 independent experiments. A *p*-value < 0.05 was considered statistically significant. Asterisks (*) indicate statistical differences between the treatments and their respective controls (*** *p* < 0.001, ** *p* < 0.01, * *p* < 0.05), plus signs (+) denote statistical differences between the treatments and 6-OHDA (+++ *p* < 0.001, ++ *p* < 0.01, + *p* < 0.05), dollar signs ($) indicate statistical difference between 6-OHDA+resveratrol and 6-OHDA+mixture of the two polyphenols or 6-OHDA and viniferin ($$$ < 0.001, $ < 0.05) and number signs (#) indicates statistical difference between 6-OHDA+viniferin and 6-OHDA + mixture of the two polyphenols (### *p* < 0.001, ## *p* < 0.01). The same symbols were used in the experiments where LPS was used instead of 6-OHDA.

## 3. Results

### 3.1. Viniferin Reduced 6-OHDA-Induced Cytotoxicity and Promoted Cell Survival

**Figure 2:**
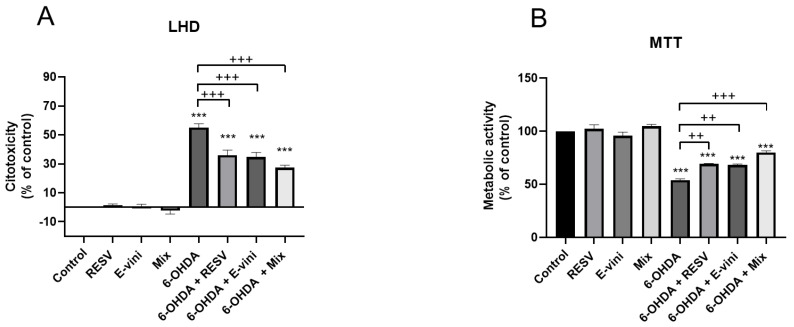
Levels of cytotoxicity (**A**) and metabolic activity (**B**) of PC12 neurons pretreated for three hours with culture medium (control), resveratrol, viniferin or a mixture of both polyphenols (Mix), all at a final concentration of 10–9. Cells were then treated with 6-hydroxydopamine (6-OHDA) at 50 μM for 24 h, as described in the Material and Methods. The supernatants and the cells were used to perform cell death (lactate dehydrogenase (LDH) assay). Data are expressed as means ± SEM of five independent experiments. For each experiment, each condition was in assessed in sextuplicate. Asterisks (*) indicate statistical differences between the treatments and their respective controls (*** *p* < 0.001), plus signs (+) denote statistical differences between 6-OHDA and 6-OHDA in the presence of polyphenols (+++ *p* < 0.001, ++ *p* < 0.01,).

To evaluate whether viniferin, alone and in combination with resveratrol protected neuronal PC12 cells from 6-OHDA-induced cytotoxicity, we investigated LDH release and metabolic activity in neuronal cells pre-treated for 3 h with resveratrol, viniferin or their combination, before administration of 6-OHDA neurotoxin for 24 h. When the two polyphenols, both at a concentration of 10–9 M, were administered alone before 6-OHDA treatment, they induced a decrease in LDH release by 19 ± 2.5% and 20.3 ± 1.3%, respectively for resveratrol and viniferin (Figure 2A) and increased cellular metabolic activity (Figure 2B) compared to cells treated with 6-OHDA only, by 15.3 ± 0.9% and 14.3 ± 1.9%, respectively. Moreover, preincubation with a mixture of resveratrol and viniferin at a final concentration of 10 -9 M, induced a 27.7 ± 2.3% decrease in LHD release compared to cells exposed to 6-OHDA only (Figure 2A). The same effect was observed for cellular metabolic activity with an increase of 26 ± 2.1% (Figure 2B), notably the effect of the mix of the two polyphenols administered before 6-OHDA challenge was more marked compared to the single polyphenols (Figure 2B).

### 3.2. Viniferin Decreased the Rate of Apoptotic Neuronal PC12 Cells

**Figure 3:**
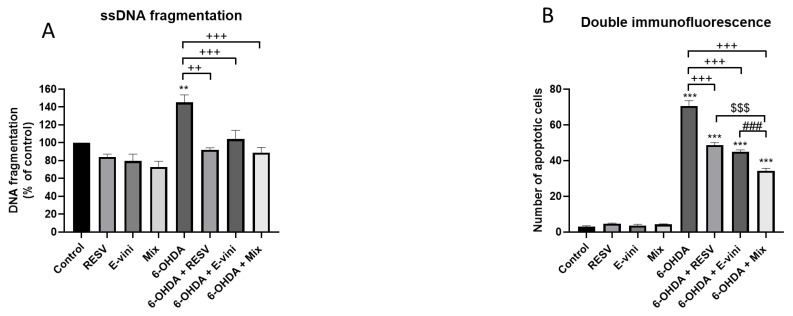
Detection of apoptosis by single stranded (ssDNA) fragmentation (**A**) and by caspase-3 and TUNEL double immunofluorescence (**B**). PC12 neurons pre-treated for three hours with culture medium (control), resveratrol, viniferin or a mixture of both polyphenols (Mix) all at a final concentration of 10–9 M. Cells were then treated with 6-OHDA at 50 μM for 24 h, as described in the Material and Methods. Apoptotic neuronal cells among 300 randomly chosen neuronal cells were counted on 10 different optical fields from 3 slides per group, as described in Material and Methods. Data are expressed as means ± SEM of three experiments. For each experiment, each condition was assessed in triplicate. Asterisks (*) indicate statistical differences between the treatments and their respective controls (*** *p* < 0.001, ** *p* < 0.01), plus signs (+) denote statistical differences between 6-OHDA and 6-OHDA in the presence of polyphenols (+++ *p* < 0.001, ++ *p* < 0.01), dollar sign ($) indicates statistical difference between 6-OHDA+resveratrol and 6-OHDA+mixture of the two polyphenols ($$$ < 0.001) and number sign (#) indicates statistical difference between 6-OHDA+viniferin and 6-OHDA + mixture of the two polyphenols (### *p* < 0.001).

In order to determine whether polyphenols may counteract cell death by inhibiting the apoptotic cascade, we assessed DNA fragmentation using a ssDNA apoptosis ELISA kit. In line with the results illustrated in Figure 2, resveratrol and viniferin alone or in combination inhibited 6-OHDA-induced DNA fragmentation in neuronal PC12 cells by 52.8 ± 9.6%, 52 ± 9.2% and 55.8 ± 10.3%, respectively (Figure 3A). This result was further confirmed by double immunofluorescence indicating an increase in TUNEL and cleaved caspase-3 positive cells after 6-OHDA treatment (Figure 3B). Notably, this anti-apototic effect was more marked when resveratrol and viniferin were used in combination which induced a 36.3 ± 4.2% decrease in the number of apoptotic cells relative to 6-OHDA treated cells, compare to the 22 ± 4% and 25.7 ± 2.3% of resveratrol and viniferin, respectively (Figure 3B).

### 3.3. Viniferin Used Alone or in Combination with Resveratrol Decreased the Cleavage of Caspase-3 and PARP-1 in Neuronal PC12

**Figure 4:**
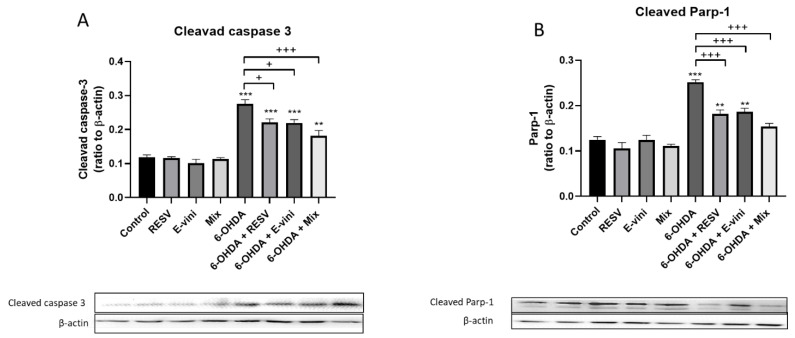
Expression of apoptotic cleaved caspase-3 (**A**) and cleaved Parp-1 (**B**) in PC12 neurons pretreated for three hours with the culture medium (control), resveratrol, viniferin or a mixture of both polyphenols (Mix) at a final concentration of 10--9 M. Cells were then treated with 6-OHDA at 50 μM for 24 h, as described in the Material and Methods. Top panel: Data are expressed as the ratio of cleaved caspase-3 (**A**) or cleaved Parp-1 (**B**) to β-actin. Bottom panel: representative Western blot bands of cleaved caspase-3, cleaved Parp-1 or β-actin. Optical densities were measured on the same membrane. Data are expressed as means ± SEM of three experiments performed in triplicate. Asterisks (*) indicate statistical differences between the treatments and their respective controls (*** *p* < 0.001, ** *p* < 0.01), plus signs (+) denote statistical differences between 6-OHDA and 6-OHDA in the presence of polyphenols (+++ *p* < 0.001, + *p* < 0.05).

To further confirm the ability of viniferin to counteract the activation of the apoptotic signaling cascade, we investigated the cleavage of caspase-3 and PARP-1, two dominant markers of early apoptotic signaling, by Western blotting. 6-OHDA increased the cleavage of both caspase-3 and PARP-1 (Figure 4A,B), while these effects were blunted by pre-incubation of neuronal PC12 cells with either viniferin (20.5 ± 0.8% for caspase-3 and 26.1 ± 1.5% forPARP-1), resveratrol (19.8 ± 2.6% for caspase-3 and 27.7 ± 3.3% for PARP-1) or their combination (34.6 ± 2.6 for caspase-3 and 38.9 ± 1.5% for PARP-1) before administration with 6-OHDA (Figure 4A,B). This effect was more pronounced when the mixture of resveratrol and viniferin was administered. In particular, the cleavage of PARP-1 did not increase in the cells pre-treated with the mix of polyphenols compared to control, despite these cells being exposed to 6-OHDA (Figure 4B). These data strongly suggest that, viniferin or resveratrol, used alone or in combination, exerted anti-apoptotic effects, confirming our results illustrated in Figure 2 and Figure 3.

### 3.4. Viniferin, Resveratrol and Their Combination Decreased Neuronal PC12 Cytotoxicity Induced by Activated Microglia, in a Co-Culture System

**Figure 5:**
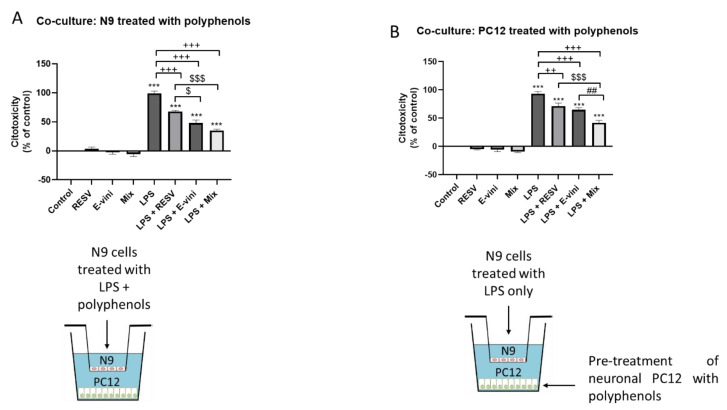
Co-culture of N9 microglial cells on neuronal PC12 cells. (**A**) N9 cells were pre-treated with polyphenols followed incubation with lipopolysaccharide (LPS), as described in the Materials and Methods. (**B**) N9 cells were treated with LPS and neuronal PC12 were pre-incubated with polyphenols as described in the Materials and Methods. Data are expressed as means ± SEM of three experiments. Asterisks (*) indicate statistical differences between the treatments and their respective controls (*** *p* < 0.001), plus signs (+) denote statistical differences between LPS and LPS in the presence of polyphenols. (+++ *p* < 0.001, ++ *p* < 0.01), dollar sign ($) indicates statistical difference between LPS + resveratrol and LPS + mixture of the two polyphenols ($$$ < 0.001, $ < 0.05) and number sign (#) indicates statistical difference between LPS + viniferin and LPS + mixture of the two polyphenols (## *p* < 0.01).

We have previously demonstrated that LPS-activated N9 microglia cells induced cytotoxicity in neuronal PC12 in a paracrine fashion in a microglia-neuronal PC12 cells co-culture system [9,11]. In order to evaluate whether viniferin may prevent N9 activation and subsequent neuronal PC12 cytotoxicity, N9 cells were pre-treated with the two polyphenols or their mixture and incubated with LPS for 24 h before being co-cultured with PC12 neurons for additional 24 h (Figure 5A). Viniferin, resveratrol and their combination counteracted the increase in LDH release from PC12 neurons co-cultured with LPS-activated N9 microglia, by 51 ± 7.8%, 31.3 ± 5.4% and 64 ± 4.4%, respectively (Figure 5A). This effect was even more marked for viniferin, and the combination of viniferin and resveratrol compared to resveratrol (Figure 5A), suggesting an anti-inflammatory role for viniferin and the mixture of viniferin and resveratrol. Furthermore, to evaluate whether these polyphenols exerted a neuroprotective effect against activated-microglia secretions, neuronal PC12 cells were pretreated with either viniferin, resveratrol or their mixture before being incubated with LPS-stimulated microglial cells (Figure 5B). Viniferin and resveratrol, both alone and in combination, decreased microglia-induced neuronal PC12 cytotoxicity as indicated by a 28.3 ± 2.7%, 21.7 ± 1.5% and 51.3 ± 2% decrease in LDH release, respectively (Figure 5B), suggesting a neuroprotective role for these polyphenols. Notably, the release of LDH was significantly lower for neuronal PC12 pre-treated with the combination of viniferin and resveratrol compared to each independent polyphenol.

### 3.5. Viniferin Affected the Secretion of Pro-Inflammatory Cytokines from LPS-Activated N9 Cells

**Figure 6:**
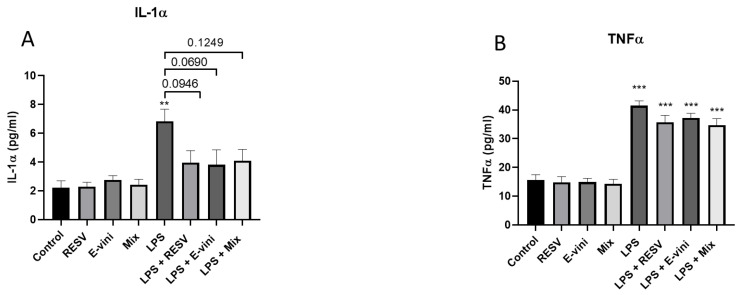
Measurements of cytokine secretion from LPS-activated microglial N9 cells by ELISA specific kit for IL-1α (**A**) and TNFα (**B**). Data are expressed as means ± SEM of four experiments. Asterisks (*) indicate statistical differences between the treatments and their respective controls (*** *p* < 0.001, ** *p* < 0.01).

To further investigate the role of viniferin, resveratrol or their mixture on neuroinflammatory mechanisms in N9 microglia-neuronal PC12 cells co-culture, we investigated whether these polyphenols may counteract LPS-induced secretion of pro-inflammatory cytokines. Figure 6 shows that a 24-h LPS treatment increased the secretion of both IL-1α (Figure 6A) and TNFα (Figure 6B). While neither viniferin, resveratrol nor their combination counteracted the secretion of TNFα triggered by LPS (Figure 6B), pre-treating N9 cells with these polyphenols tended to decrease the levels of IL-1α in the cell culture media compared to cells exposed to LPS only (43.3 ± 7.3% for resveratrol; 46.7 ± 11.1% viniferin; 41.5 ± 6.3% for their combination) (Figure 6A).

## 4. Discussion

This study provides evidence on the neuroprotective and anti-inflammatory potential of the polyphenol viniferin in a cellular model of PD, PC12 dopaminergic neurons, and N9 microglia–PC12 neurons co-culture system [9,10]. These effects were underlie by the ability of this polyphenol to counteract the activation of the apoptotic cascade induced by 6-OHDA in neuronal cells and inhibit cytotoxicity induced by LPS-activated microglia in a microglia–neuron co-culture system. Although our in vitro model could not perfectly replicate in vivo neuron–microglia physiology, these results enlighten that when viniferin was mixed with its homologous resveratrol, it also proved to be more effective in countering neurotoxicity induced by pro-inflammatory activated N9 microglia. Remarkably, despite the fact that final concentration of the mixture of the two polyphenols matched the concentration of the single polyphenols used in isolation, its effect was more powerful in preventing 6-OHDA-induced increase in caspase-3/TUNEL-positive PC12 neurons and inhibiting cytotoxicity promoted by LPS-activated N9 microglia, than resveratrol or viniferin alone.

6-OHDA, due to its similarities to endogenous catecholamines, is taken up and accumulates in catecholaminergic neurons promoting neurotoxicity mainly via oxidative stress-related mechanisms [28]. In support of this, its administration directly into the brain via stereotactic surgery induces parkinsonism in rodent models by promoting nigrostriatal pathway degeneration [29]. These effects were recapitulated in the present study in which 6-OHDA promoted cytotoxicity, as indicated by an increase in LDH release from PC12 dopaminergic neurons and a decrease in their metabolic activity. Importantly, these neurotoxic effects are dependent on the activation of apoptosis as indicated by an increase in caspase-3/TUNEL-positive cells, DNA fragmentation and the cleavage of caspase-3 and PARP, as our present results illustrate.

Nonetheless, despite the deleterious impact of 6-OHDA on cell viability, it was sufficient to administrate the PC12 neuronal cells with resveratrol, viniferin or their combination to inhibit 6-OHDA-induced apoptosis. Of these polyphenols, resveratrol has been widely described for its antioxidant properties [30,31,32,33] which are mainly dependent on its ability to induce key transcription factors such as nuclear factor erythroid 2-related factor (Nrf2) and antioxidant enzymes, including glutathione S-transferase (GST) [33,34], all crucial antioxidants defenses [35]. Given the role of oxidative stress in the pathogenesis of neurodegenerative diseases, it represents a promising target to counter neuronal loss [36]. Indeed, resveratrol has been reported to exert neuroprotective effects both in vitro and in vivo [15,37,38,39,40,41], which are in agreement with the results reported herein, most likely dependent on the ability of resveratrol to dampen 6-OHDA-induced oxidative stress triggered by mitochondrial dysfunction [42]. We also investigated in our cell culture system whether a similar effect could be exerted by the naturally occurring resveratrol dimer, viniferin, and if these molecules could act synergically. We report that viniferin was able to inhibit 6-OHDA-induced neurotoxicity and apoptosis to the same extent as resveratrol. These effects, in parallel to those exerted by resveratrol, may be dependent on the antioxidant properties of viniferin [43]. Furthermore, the results reported as part of this study are corroborated by previous evidence supporting the neuroprotective role of viniferin, not only in ADmodels [18,44], but also in PD cellular models [45]. In this regard, in a PD cell model represented by a human neuroblastoma SH-SY5Y cell line exposed to rotenone, viniferin reduced cellular apoptosis, oxidative stress and restored mitochondrial homeostasis, all underlain by SIRT3-mediated fork headbox O3 (FOXO3) deacetylation [45], with FOXO3 playing an important role in PD progression and dopaminergic neuron survival [46]. While this study further supports our results and provide mechanistic insights into the role of viniferin, our data highlight the potency of viniferin considering it was able to inhibit apoptosis at a concentration of 10 nM compared to previous reports using 1 µM for 24 h [45]. Remarkably, the ability of viniferin to counteract apoptosis appeared to be potentiated by resveratrol, with these two molecules acting jointly to increase cellular metabolic activity and decrease the number of caspase-3/TUNEL-positive cells following exposure to 6-OHDA, in our cellular paradigm. Even if the results from cellular paradigms cannot be directly transferred in vivo, we believe that the combination effect of these two molecules represents a promising nutraceutical combination to investigate in in vivo models of PD.

Inflammation represents another pivotal pathogenetic mechanism in PD, underpinned by microglia over-activation which contributes to dopaminergic neuron loss [8,47,48,49]. In fact, intranigral injection of LPS, a known pro-inflammatory component of Gram-negative bacteria, promotes dopaminergic neuron death recapitulating the pathogenetic features of PD in rodent models [50,51]. This is corroborated with our co-culture model where LPS-activated N9 microglia cells promoted cytotoxicity in downstream neuronal PC12 cells in the absence of a direct cell-to-cell contact, further supporting the role of inflammation in promoting neurotoxicity and its involvement in PD pathogenesis. Furthermore, in consideration of the lack of a direct contact between N9 cells and neurons in our co-culture system, the neurotoxic effect elicited by microglia is mediated by its secretory milieu. Indeed, the activation of the Toll like receptor 4 (TLR4) by LPS in microglial cells triggers the activation of pro-inflammatory pathways, including nuclear factor κB (NFκB) which promotes the secretion of pro-inflammatory cytokines, ROS and nitric oxide via the activation of NADPH oxidase and inducible nitric oxide synthase, respectively [52]. These cytotoxic factors, may also represent the mediators linking microglia activation with PC12 neurons cytotoxicity [53], as indicated in our data by an increase in IL-1α and TNF-α secretion by N9 microglia cells upon exposure to LPS. Remarkably, we demonstrated that resveratrol, viniferin or their combination was able to counteract microglia-induced neurotoxicity both indirectly when these polyphenols were applied to N9 cells, as well as directly when they were used to treat PC12 neurons. This indicates that resveratrol and viniferin were both able to modulate microglia secretory milieu, reducing the cytotoxic effect of microglia secretome by eliciting adaptive protective responses directly in neurons. In either case, resveratrol and viniferin exert anti-inflammatory effects and trigger molecular mechanisms aimed at protecting cells against inflammation and oxidative stress, possibly relying on the activation of the transcription factor nrf2 and the deacetylating enzyme SIRT-3 [17,34]. Particularly, in microglial cells, the activation of nrf2 by resveratrol may be responsible for the inhibition of NFκB via a cross-talk between these transcription factors [54], leading to the consequent downregulation of the inflammatory response elicited by LPS. Similarly, viniferin has also been shown to exert anti-inflammatory responses by inhibition NFκB signaling [55]. Thus, the ability of these polyphenols to restrain activated-microglia from promoting cytotoxicity in PC12 neurons may be dependent on their capacity to dampen LPS-induced inflammation and the consequent release of neurotoxic factors from N9 cells, including nitric oxide, ROS and cytokines. Nonetheless, despite cytokines being a potential driver of microglia-induced neurotoxicity, they are not significantly downregulated by resveratrol or viniferin in our co-culture system. In light of this, these polyphenols may also modulate N9 secretion of neurotoxic mediators such as ROS, nitric oxide and reactive nitrogen species [6,13,52,56,57] that, however, were not measured in our study. Resveratrol and viniferin also exerted direct neuroprotective effect on PC12 neurons co-cultured with activated microglia. This action may rely on the activation of a transcriptional reprogramming triggered by polyphenols in PC12 neurons in order to induce genes involved in cellular defenses, which may be dependent on nrf2 and SIRT-3 activation [17,34]. However, this paradigm remains to be fully elucidated.

To conclude, these polyphenols not only directly protect PC12 neurons against 6-OHDA, but also exert their neuroprotective properties by inhibiting the neurotoxic effect promoted by LPS-activated microglia. Moreover, the neuroprotective potential of resveratrol and viniferin is potentiated when they are used in combination, suggesting a synergistic effect. Thus, considering that the foodstuff rich in resveratrol also contains other forms of stilbenes, including viniferin, and taking into account the neuroprotective potential of their combination, the consumption of these foods may represent a valuable nutritional intervention in complementary therapies for neurodegenerative diseases.

## Data Availability

All data are available upon reasonable request to M.-G. Martinoli.
